# Supplementary material for: Memristor-based storage system with convolutional autoencoder-based image compression network
Source: Nat Commun. 2024 Feb 7;15:1132. doi: 10.1038/s41467-024-45312-0 (PMC10850548; doi:10.1038/s41467-024-45312-0)
Supplement: Supplementary file 1 — Supplementary Information [file 41467_2024_45312_MOESM1_ESM.docx]

**Memristor-based storage system with** **convolutional autoencoder-based image compression network**

Yulin Feng^1,2^, Yizhou Zhang^1^, Zheng Zhou^1^, Peng Huang^1*^, Lifeng Liu^1*^, Xiaoyan Liu^1^, and Jinfeng Kang^1^

^1^School of Integrated Circuits, Peking University, Beijing 100871, China.

^2^Key Laboratory of the Ministry of Education for Optoelectronic Measurement Technology and Instrument, Beijing Information Science & Technology University, Beijing 100192, China.

^*^Corresponding authors. Emails: phwang@pku.edu.cn; lfliu@pku.edu.cn


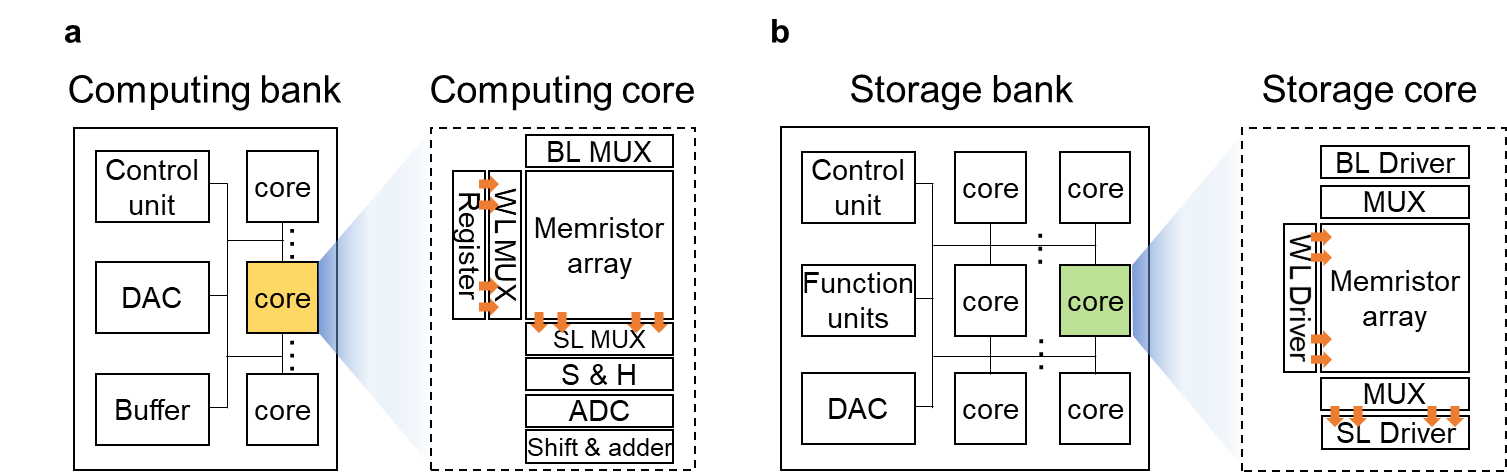


**Supplementary Figure 1 |** **Diagram of memristor-based near-storage in-memory processing architecture.** **a,** Computing bank and a memristor-based computing core. DAC, ADC, and S & H refer to the digital-to-analog converter, analog-to-digital converter, and sample & hold, respectively. WL, BL, and SL denote the word line, bit line, and source line, respectively. **b,** Storage bank and internal structure of a storage core.


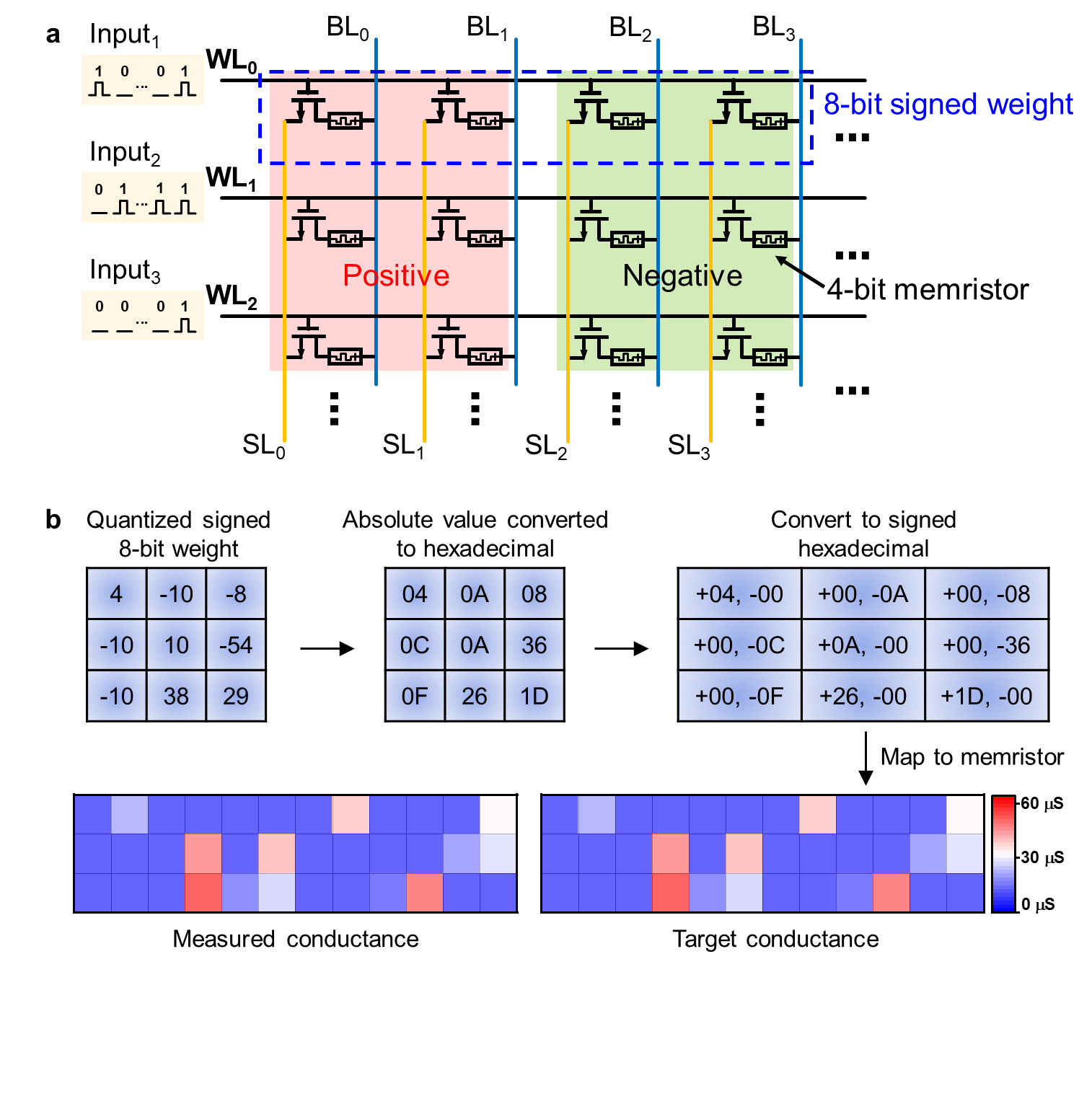


**Supplementary Figure 2 | Weight mapping scheme.** **a,** Mapping of signed 8-bit kernel weights into 4-bit memristor arrays. The 4-bit memristor has sixteen conductance states and thus can represent one hexadecimal digit. In this case, we can use two 4-bit memristors to represent one 8-bit weight. For example, decimal 4 is converted into hexadecimal as 04, representing 16^1^×0 + 16^0^×4 = 4. Hence, each signed weight can map to four memristor columns: two columns for positive weights with positive coefficients on output and the other for negative weights with negative coefficients on output. **b,** Typical kernel weights mapping process. After training in the software, the 32-bit floating-point weights are quantized to signed 8-bit. Next, the weights are converted to two hexadecimal numbers. Considering the sign of the weights, if the weight is positive, it will be mapped to the positive two columns, and the negative two columns remain zero (ultra-low conductance). By precise programming, the weights in software are transferred into memristor arrays.

**Supplementary Figure 3 |** **Area overhead of a computing core for different memristor array configurations.** Different colors represent different circuit modules.


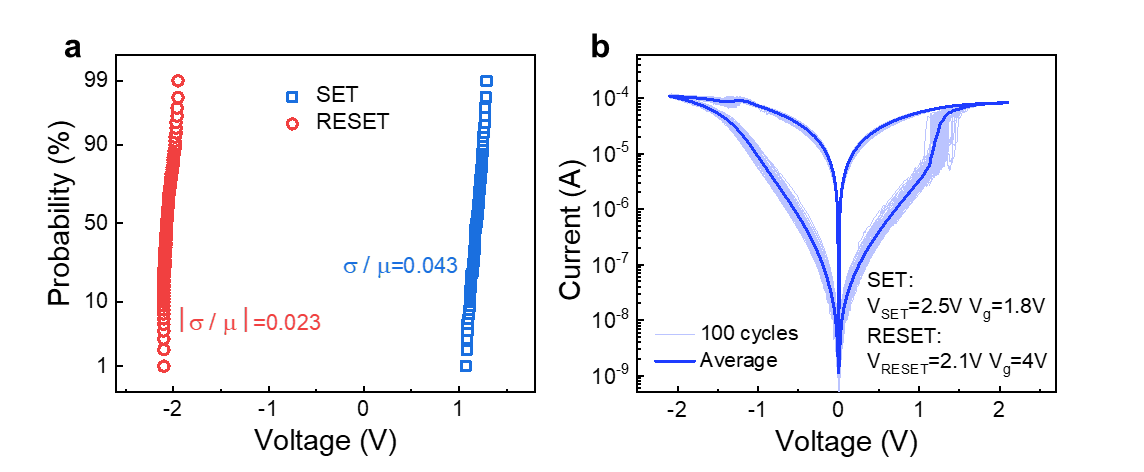


**Supplementary Figure 4 | Electrical properties of the memristor array.** **a,** Statistical distribution of the SET and RESET voltages of random selected 100 cells in memristor array. σ and μ refer to the standard deviation and mean, respectively. **b,** The quasi-static direct current (DC) behaviors of 100 cycles within one memristor cell.


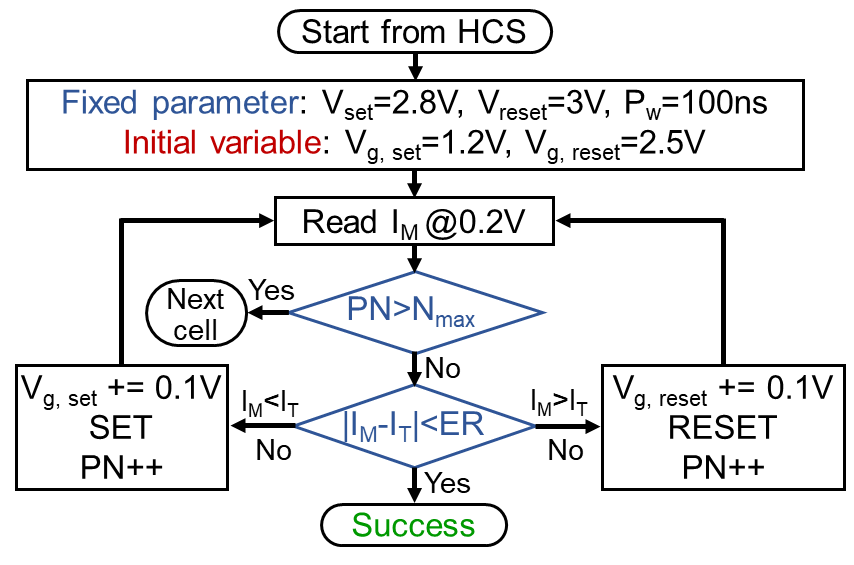


**Supplementary Figure 5 | The write-verify programming scheme.** The programming starts from high conductance state (HCS). PN refers to the pulse number. ER represents the error range, which is defined as 1 μS during programming. I_M_ denotes the current through the memristor, while I_T_ indicates the current converted from target conductance. N_max_ is set to 100.


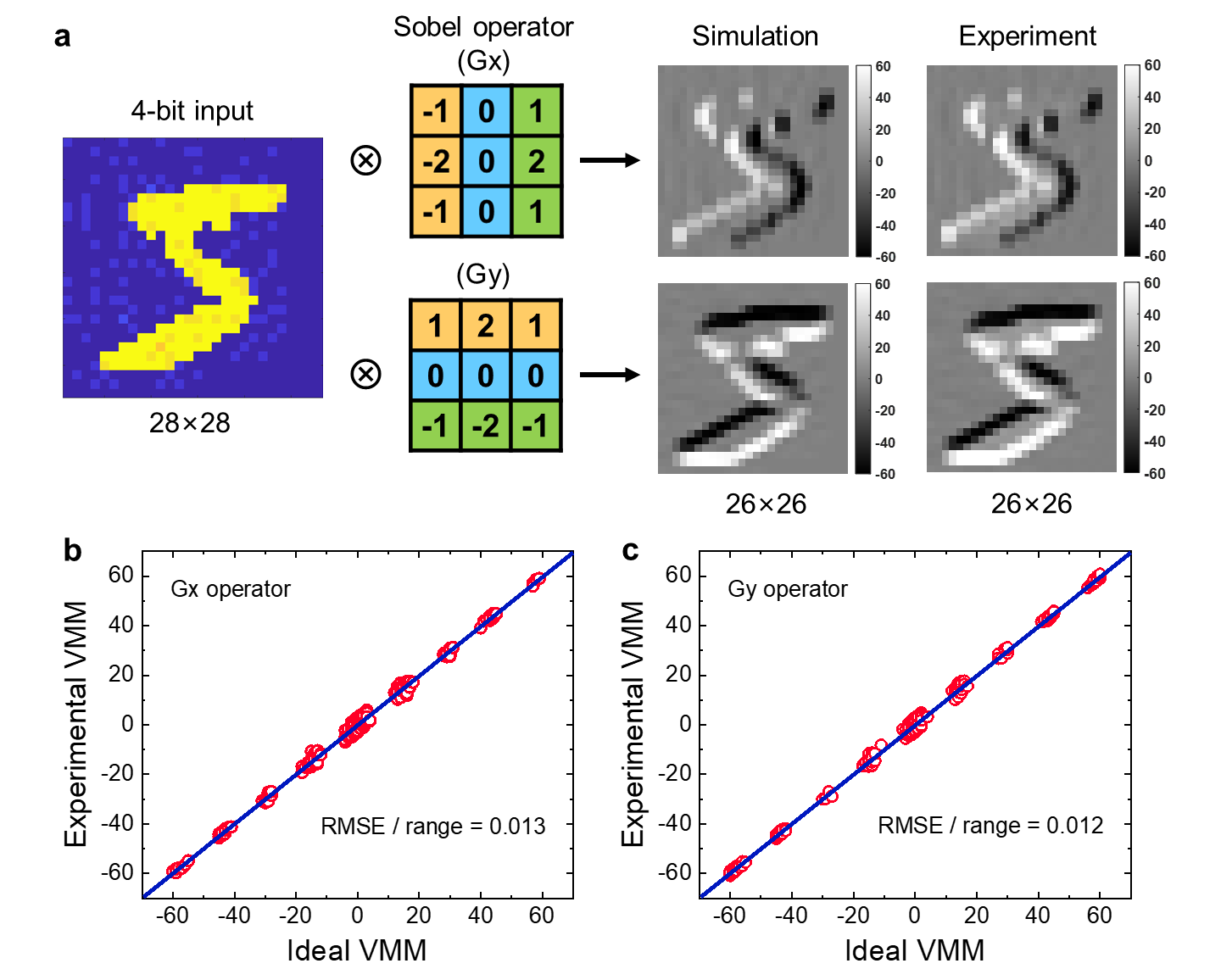


**Supplementary Figure 6 | Verification of the operation and mapping schemes.** **a,** A convolution example is performed for edge extraction based on the memristor array. The grayscale image is first converted into 4-bit for input. Sobel operators, which include G_x_ for vertical edges and G_y_ for horizontal edges, are used for edge extraction of the hand-written digit 5. The similarity of results between the simulation and experimental demonstrates the feasibility of the operation and weight mapping schemes proposed in this study. Experimental vector-matrix multiplication (VMM) results of **b,** G_x_ operator and **c,** G_y_ operator with respect to ideal results, respectively.


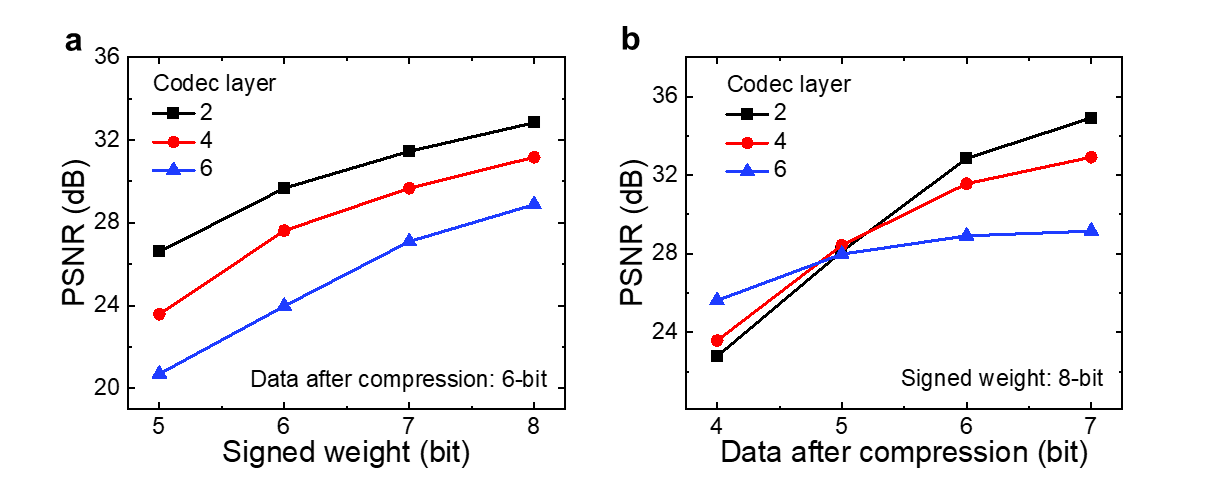


**Supplementary Figure 7 | Impact of direct quantization on convolutional autoencoder (CAE) network performance.** **a,** The peak signal-to-noise ratio (PSNR) value of the reconstructed image as a function of quantized signed weight (in this case, the compressed data is fixed at 6-bit, and other activation is fixed at 8-bit). **b,** The PSNR value with respect to the quantized compressed data (in this case, both the weight and activation are fixed at 8-bit). Different network structures are considered.

**Supplementary Figure 8 |** **Design space exploration with different weight and compressed data precisions.** CAE networks with two-, four-, and six-layer are used. During evaluation, the weight precision changes from 5-bit to 9-bit and the compressed data varies from 4-bit to 7-bit. 32 dB is set as the lower limit at which the human eye has difficulty distinguishing between original and reconstructed images. L2-C4b corresponds to a system with two-layer CAE network and 4-bit compressed data precision. After training the CAE network with different structures in software, the weights and data after compression are directly quantized with different precisions. PSNR value is used as a criterion to indicate the quality of the quantized image. It can be seen from the results that the PSNR value increases significantly as the weight quantization accuracy increases and tends to be saturated from 8-bit to 9-bit. Similarly, as the compressed data accuracy increases, the PSNR value firstly increases from 4-bit to 6-bit and then tends to be saturated from 6-bit to 7-bit. Considering the area overhead of the memristor-based storage array, we choose 8-bit precision for weight and 6-bit precision for compressed data for subsequent simulation optimization and hardware demonstration.


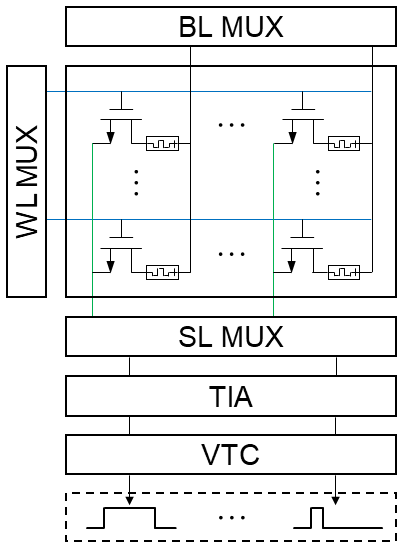


**Supplementary Figure 9 |** **Diagram of architecture for analogue readout scheme.** Architecture of memristor-based core for implementing analogue width-modulated pulses conversion. TIA and VTC refer to trans-impedance amplifier and voltage-to-time converter, respectively.


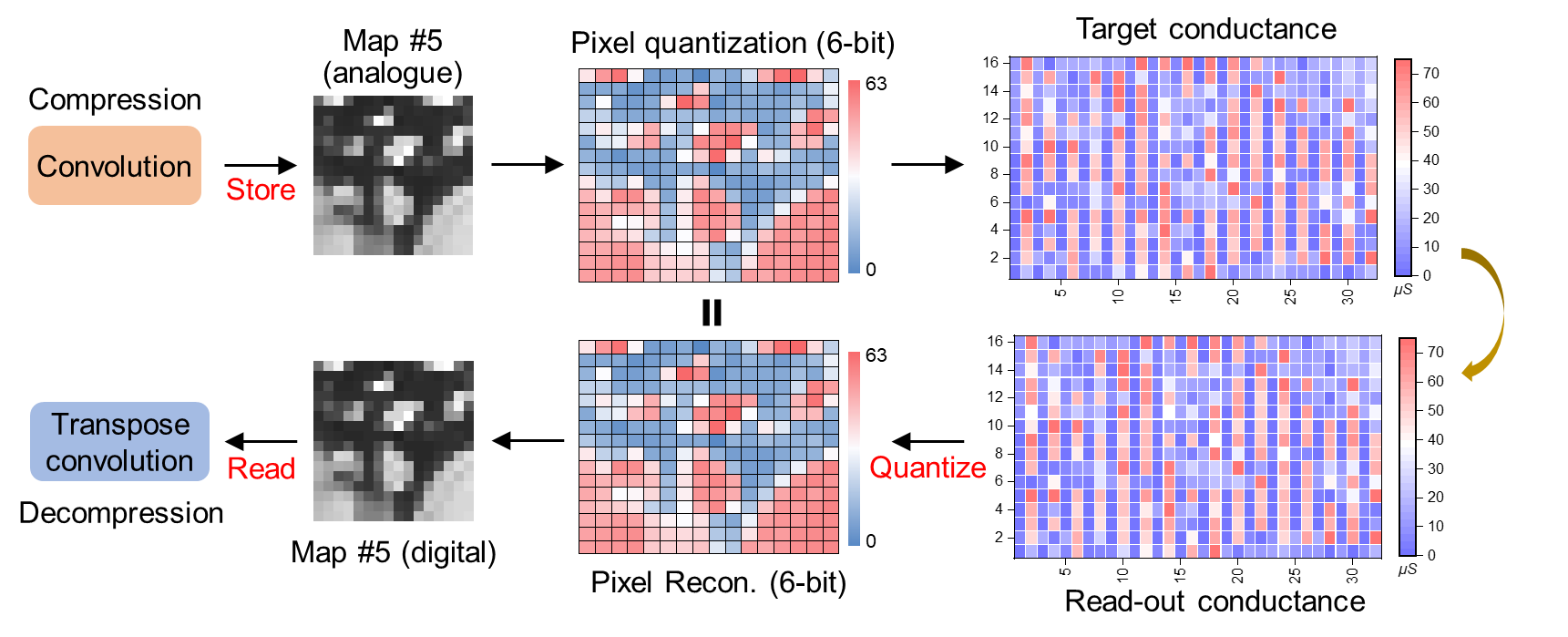


**Supplementary Figure 10 | Process of data storage and readout.** A typical process of storing and reading the compressed feature map #5 is demonstrated by using the proposed digital readout method. Different from the analogue compressed feature map just after convolution, the feature map used for the input of transpose convolution after being read from the storage array is digital. This is one of the main reasons for the pixel value deviation of the reconstructed image. Pixel Recon. means pixel reconstruction.


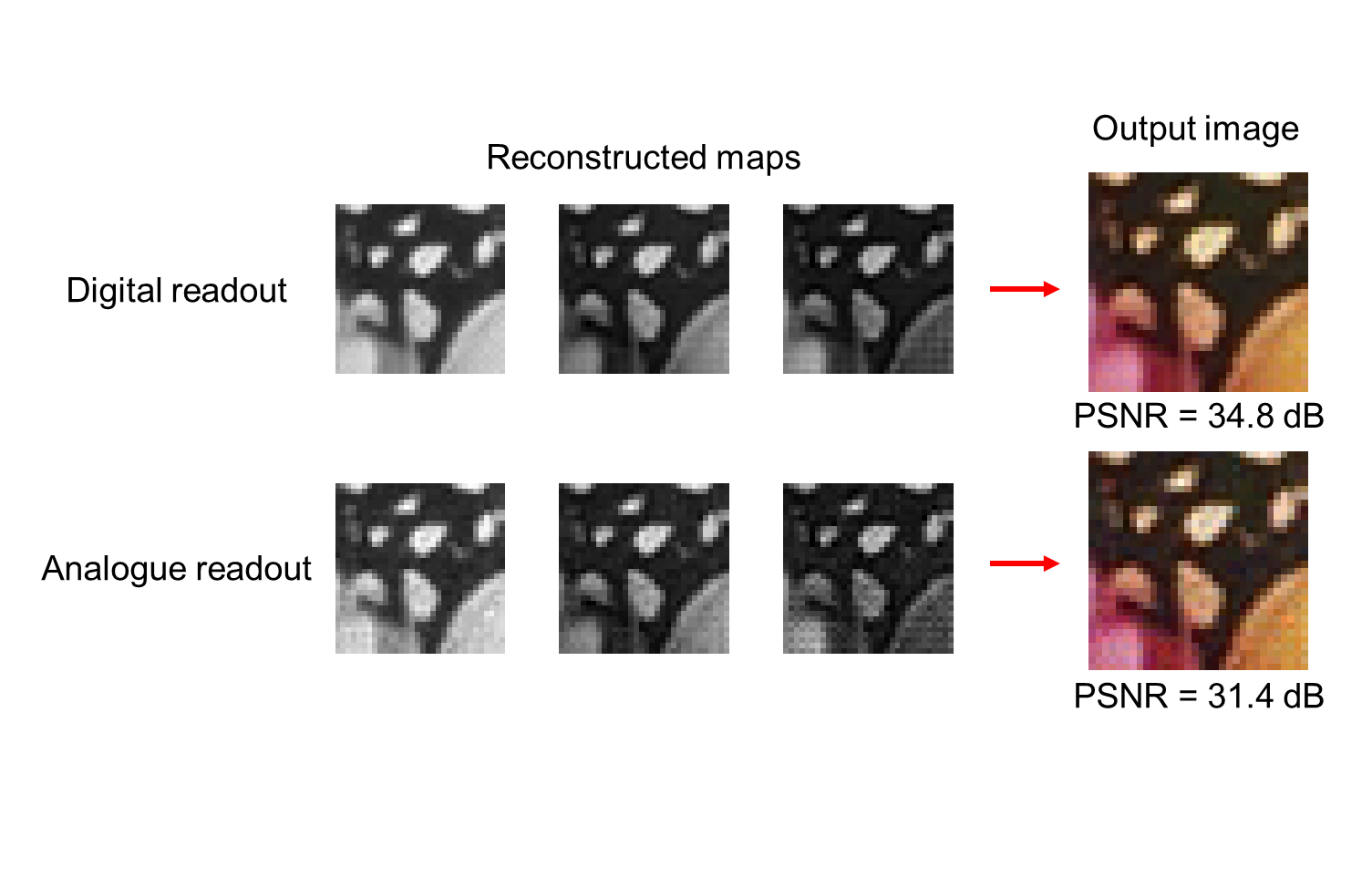


**Supplementary Figure 11 | Visualization and PSNR values of reconstructed image by using two readout methods.** Conductance fluctuation is introduced into the decompression process with analogue readout, resulting in the periodic noise appearing in the reconstructed greyscale feature maps, which ultimately deteriorates the pixel values of the output image. This phenomenon can be dramatically mitigated by using the digital readout method.


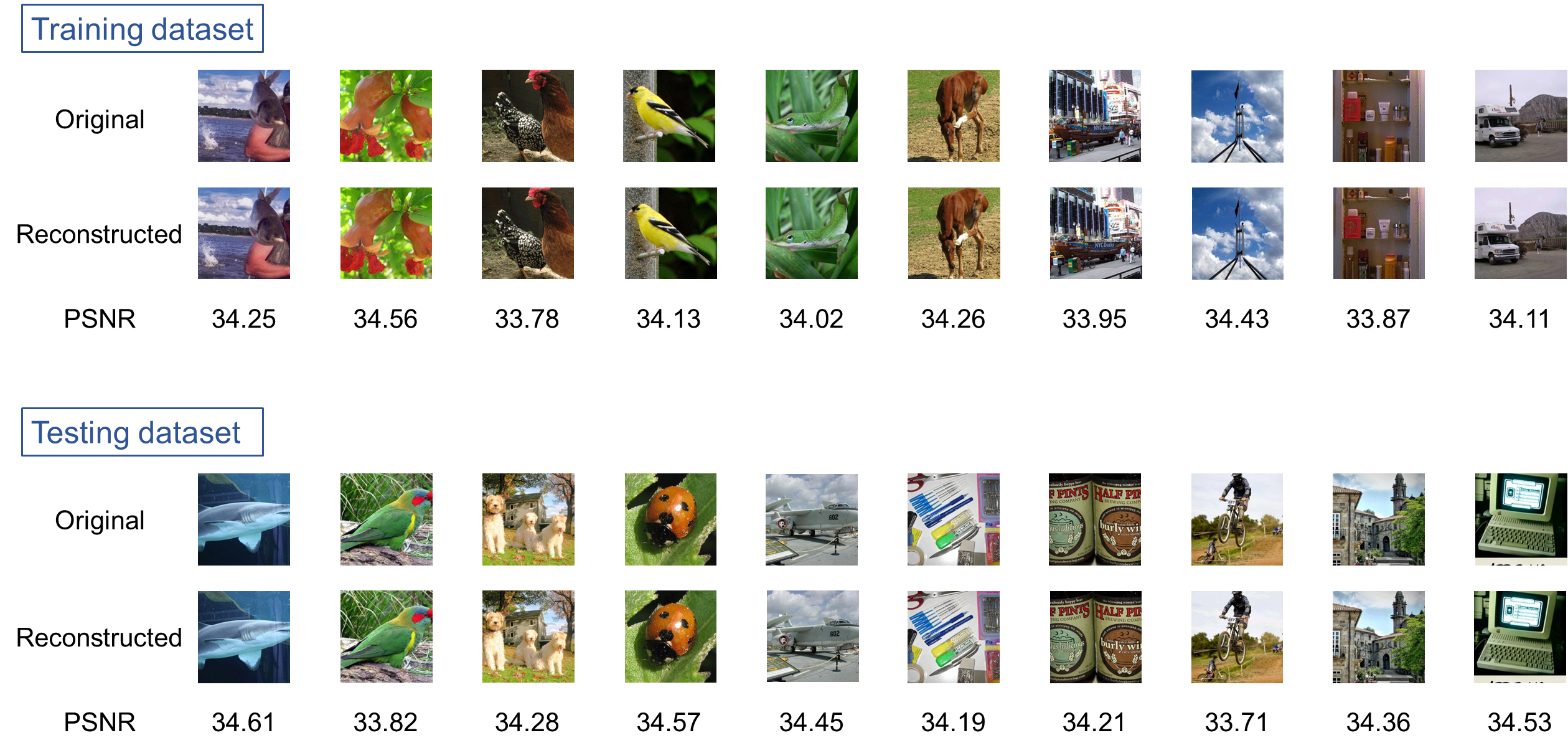


**Supplementary Figure 12 | Comparison of sample images before and after compression.** The images are randomly selected from the original and reconstructed ImageNet datasets.


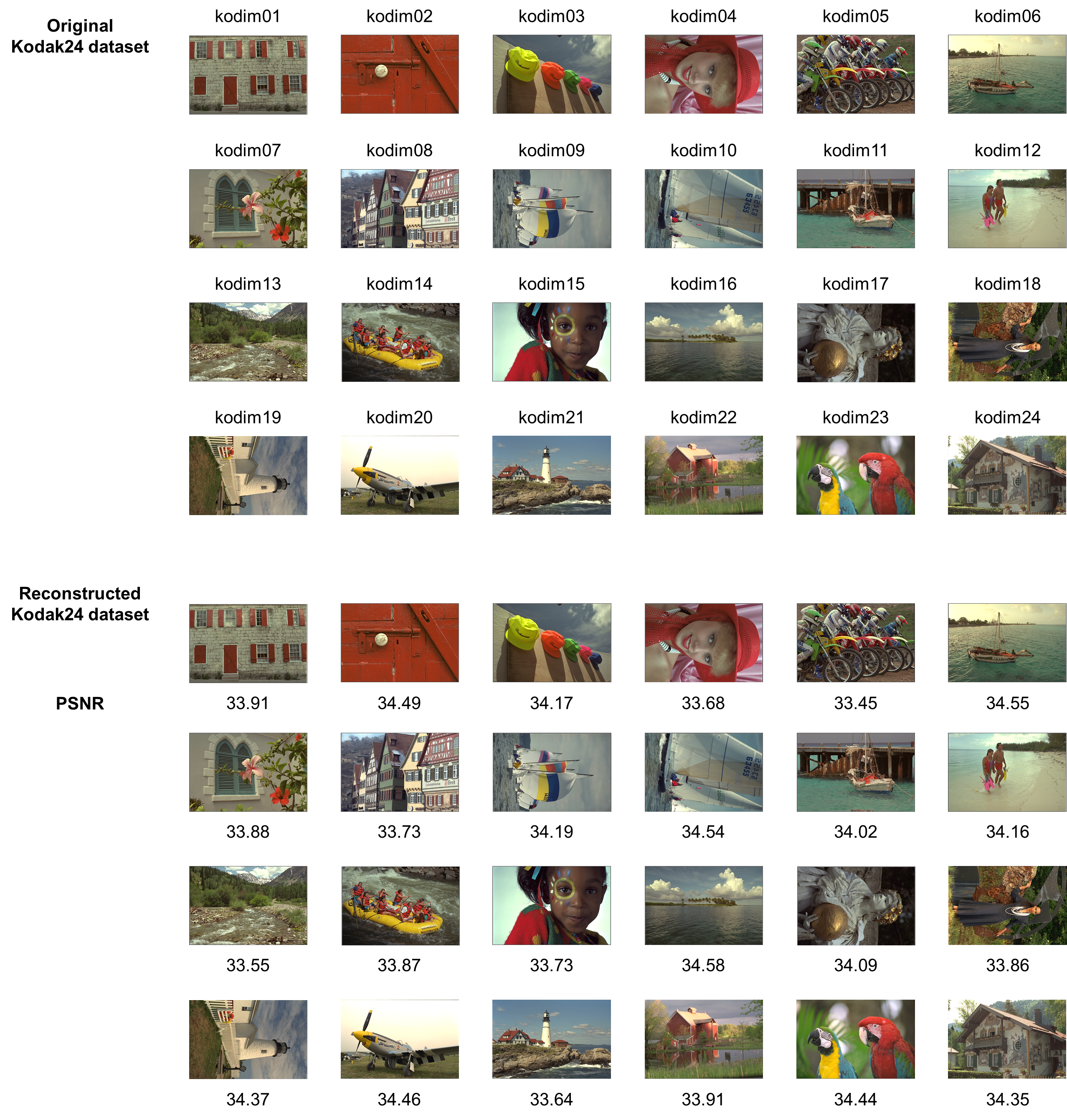


**Supplementary Figure 13 | Original and reconstructed Kodak24 dataset with PSNR values.** All the images from kodim01 to kodim24 are compressed and decompressed by using the two-layer CAE network integrated storage system with optimized weight and compressed data precisions. Each image is in RGB format with 768×512 resolution.


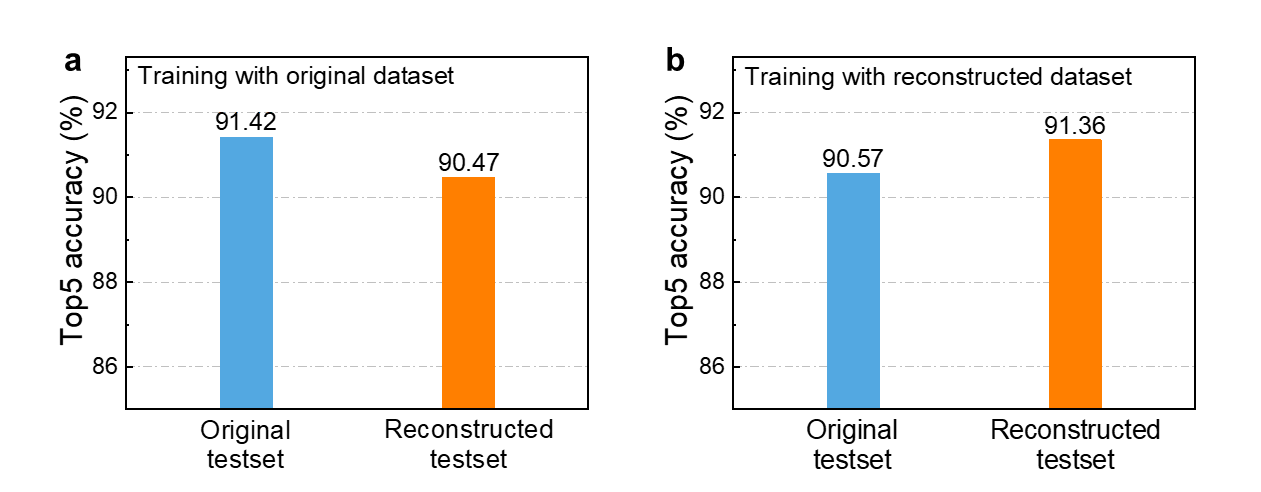


**Supplementary Figure 14 | Top5 recognition accuracy of ResNet34 network.** The network is trained with **a,** Original and **b,** Reconstructed ImageNet dataset, and then tested with both the original and reconstructed test datasets.


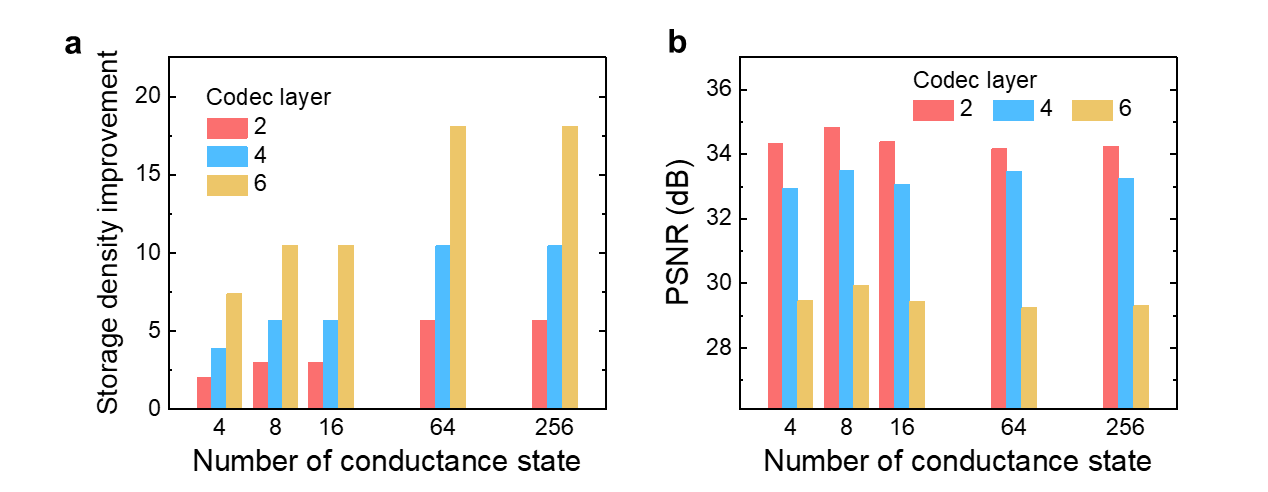


**Supplementary Figure 15 |** **Effect of memristor conductance state numbers on compression performance.** Relationships of **a,** Storage density improvement and **b,** PSNR value with respect to different memristor conductance state numbers. The simulated results show increased storage density with the increased conductance states. That is, more conductance states mean fewer memristors required for storing the same amount of data, which is conducive to improving storage density. In addition, it can be observed that when the memristor conductance state varies from 3-bit to 4-bit or 6-bit to 8-bit, the storage density improvement is almost unchanged. This is because each compressed 6-bit data is designated to be stored in an integer number of cells rather than storing multiple compressed data information within one cell, which could minimize the complexity of peripheral circuitry used for readout data processing. Meanwhile, the PSNR values remain unchanged, which are insusceptible to the variation of the storage capacity of the memristor. The results demonstrate that the memristor arrays with more conductance states can improve the storage density without concerning about performance degradation.

**Supplementary Figure 16 |** **The influence of memristor variations in different parts of the memristor-based storage system**. In the system, both the compress/decompress process and data storage/readout process are implemented in memristor arrays. Compared with the conductance fluctuation of the stored compressed data, the variation of the synapse weights has a greater impact on the network. Quant. means quantized.


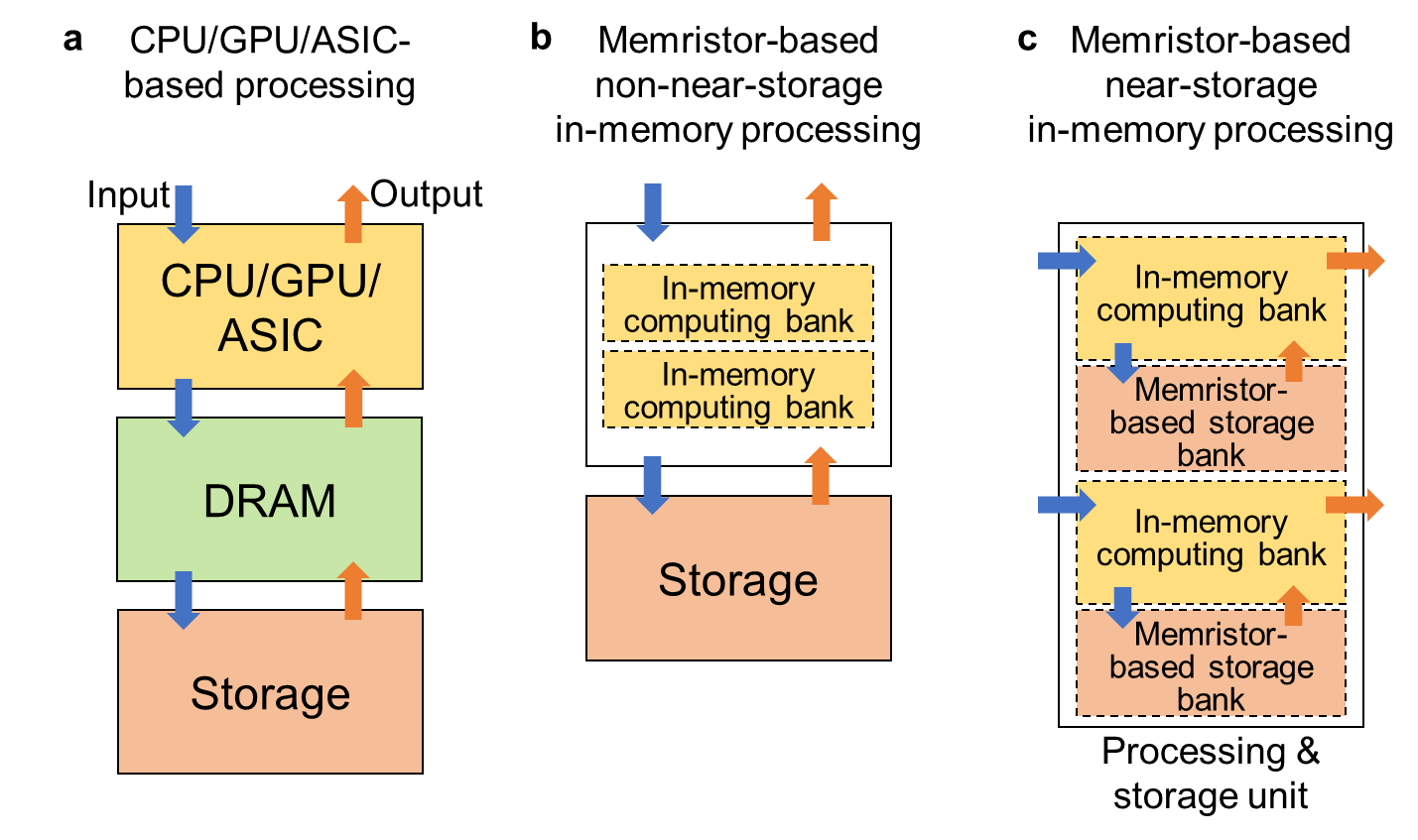


**Supplementary Figure 17 |** **System architectures used for data compression and retrieval.** Schematic diagram of **a,** Central processing unit (CPU)/graphics processing unit (GPU)/application-specific integrated circuit (ASIC)-based processing system. DRAM refers to dynamic random-access memory. **b,** Memristor-based non-near-storage in-memory processing system, and **c,** Memristor-based near-storage in-memory processing system architectures. The memristor-based computing bank and storage bank in near-storage in-memory system are located in the same physical unit, which reduces data transfer and improves processing and storage efficiencies.


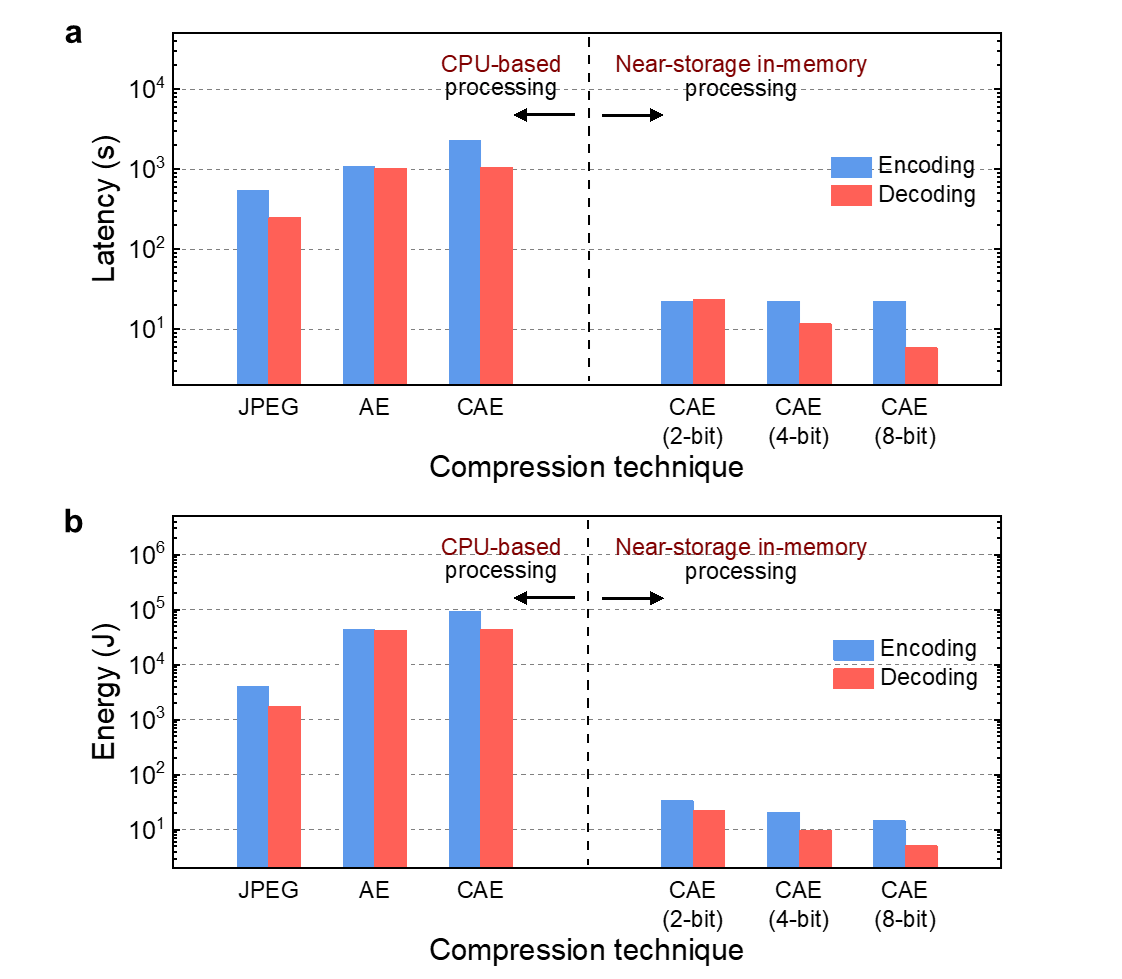


**Supplementary Figure 18 |** **Overhead comparison results of server-grade CPU-based processing system and memristor-based near-storage in-memory processing system.** **a,** Latency and **b,** Energy consumption with respect to different compression techniques and memristor multi-bit capabilities. The data compression and retrieval processes of joint photographic experts group (JPEG), autoencoder (AE), and CAE techniques are assessed under server-grade CPU-based processing approach. For near-storage in-memory processing, the storage system with 2-, 4-, and 8-bit memristor multi-bit capability is discussed using CAE network for compression and decompression.

**Supplementary Figure 19 |** **Area estimation with memristor-based near-storage in-memory processing system and server-grade CPU-based processing system.** Different memristor multi-bit capabilities and CAE network structures are used for evaluation.

**Supplementary Figure 20 |** **Statistical pulse numbers to program 4-bit memristors.** The results are obtained based on the programming scheme shown in Supplementary Fig. 5. Programming pulses of 100 cells are counted for each conductance state.

**Supplementary Table 1. Area metrics of each circuit components in one computing core with 4-kb (256×16) memristor array.**

| Module | Area (μm^2^) |
| --- | --- |
| Register | 22856 |
| WL-MUX | 21504 |
| Array | 9646 |
| SL/BL-MUX | 6208 |
| S & H | 2080 |
| ADC | 560000 |
| Shift & adder | 185600 |

**Supplementary Table 2. Latency of server-grade CPU-based processing system.**

| Compression technique | Process | CPU + DRAM (s) | Storage (s) |
| --- | --- | --- | --- |
| JPEG | Encode | 535.98 | 42.12 |
|  | Decode | 245.73 | 42.12 |
| PNG | Encode | 2072.07 | 240.33 |
|  | Decode | 344.28 | 240.33 |
| AE | Encode | 1066.87 | 27.29 |
|  | Decode | 1028.09 | 27.29 |
| CAE | Encode | 2267.26 | 124.8 |
|  | Decode | 1061.15 | 124.8 |

**Supplementary Table 3. Energy consumption of server-grade CPU-based processing system.**

| Compression technique | Process | CPU + DRAM (J) | Storage (J) |
| --- | --- | --- | --- |
| JPEG | Encode | 3884.74 | 212.99 |
|  | Decode | 1550.71 | 212.99 |
| PNG | Encode | 15171.14 | 1210.05 |
|  | Decode | 2520.63 | 1210.05 |
| AE | Encode | 43491.58 | 136.87 |
|  | Decode | 41910.66 | 136.87 |
| CAE | Encode | 92808.36 | 626 |
|  | Decode | 43437.22 | 626 |

**Supplementary Table 4. Latency and energy consumption of GPU-based processing system.**

| Performance | Process | GPU + DRAM | Storage |
| --- | --- | --- | --- |
| Latency (s) | Encode | 115.97 | 124.8 |
|  | Decode | 122.8 | 124.8 |
| Energy (J) | Encode | 1275.67 | 626 |
|  | Decode | 1350.85 | 626 |

**Supplementary Table 5. Latency and energy consumption of ASIC-based processing system.**

| Performance | Process | ASIC | DRAM | Storage |
| --- | --- | --- | --- | --- |
| Latency (s) | Encode | 0.98 | 24.41 | 124.8 |
|  | Decode | 0.43 | 24.41 | 124.8 |
| Energy (J) | Encode | 0.41 | 9 | 626 |
|  | Decode | 0.18 | 9 | 626 |

**Supplementary Table 6. Summary of metrics of key circuit modules in memristor-based computing bank used for evaluation.**

| Module | Energy | Latency | Area (μm^2^) |
| --- | --- | --- | --- |
| ADC | 14.3 pJ/bit | 2.2 ns/op | 35000 |
| DAC | 6.8 pJ/conversion | 50 ps/op | 72000 |
| Buffer | 0.38 pJ/bit | 1.04 ns/access | 1360000 |
| Array | 9.2 fJ/cell (encoding)  8.2 fJ/cell (decoding)  17 fJ/cell (reading)  25 pJ/cell (writing) | 20 ns/pulse @ reading  100 ns/pulse @ writing | 9646 @ 4-kb |

**Supplementary Table 7. Evaluation results of latency and energy during data compression and retrieval with memristor-based near-storage in-memory processing system.**

| Performance | Process | Buffer | Array | ADC | Data  write/access | Overall |
| --- | --- | --- | --- | --- | --- | --- |
| Latency (s) | Encoding | 0.27 | 10.41 | 0.57 | 22.14 | 22.14 |
|  | Decoding | 0.3 | 11.71 | 0.64 | 0.67 | 11.71 |
| Energy (J) | Encoding | 1.37 | 0.13 | 7.63 | 11.66 | 20.79 |
|  | Decoding | 0.46 | 0.04 | 8.58 | 0.7 | 9.78 |

**Supplementary Table 8. Area metrics of each circuit components in one storage core with 1-Mb (1024×1024) memristor array.**

| Module | Area (μm^2^) |
| --- | --- |
| WL driver | 155.6 |
| Array | 53162 |
| SL/BL driver | 413.6 |
| SL/BL-MUX | 2998.6 |

**Supplementary Note I. Bit-slicing encoding technique**

During the hardware demonstration of the convolutional autoencoder (CAE) network, we utilized the bit-slicing encoding technique to process the input data. By using this technique, the pixel information (RGB image or compressed data) of the input image is converted into different bits from the most significant bit (MSB) to the least significant bit (LSB) with binary format which can correspond to binary pulse. Next, binary pulses are input to the gate of the transistor (WL) in 1-transistor 1-memristor (1T1M) array in sequence. The high level (5V) of the pulse represents input logic 1, meaning the transistor is on, while the low level (0V) represents input logic 0 and the transistor is off. The current readout from the source line (SL) is the result of the multiply and accumulate (MAC) operation.

**Supplementary Note II. Memristor array structure design**

When designing the structure of 1T1M array, fewer columns’ design means reduced number of SL/bit line (BL)-MUX, analog-to-digital converter (ADC), sample and hold (S & H), and shift & add modules in peripheral circuitry of in-memory computing core if the size of the 1T1M array is fixed. Although it will increase the number of word line (WL)-MUX and Register modules, the area overhead would decrease since the area of the circuit connected to SL/BL is more dominant than the area of the circuit connected to WL. Based on the parameters of each circuit module listed in Supplementary Table 1 and Table 6, we compared the area overhead of the peripheral circuitry under three array structures of 256×16, 128×32, and 64×64, as shown in Supplementary Fig. 3. The result implies that the 1T1M array with 256×16 configuration occupies the least area. In addition, the array with 256×16 can process vector-matrix multiplication (VMM) with a maximum parallelism of 256 in WL. So, the throughput is the same as the other configuration designs, even though the calculation parallelism of SL decreases.

Based on the above considerations, we have chosen a 256×16 array configuration for the integration of memristors which are used for in-memory computing.

**Supplementary Note III. Conversion of analogue width-modulated pulse**

The conversion of multi-bit conductance readout from the memristor array into analogue width-modulated pulses can be designed as the architecture shown in Supplementary Fig. 9. The current readout from the memristor array passes through a trans-impedance amplifier (TIA) to convert the current signal into the voltage signal. Then, the voltage-to-time converter (VTC) is adopted to convert the analogue input voltage into an analogue pulse. The overhead of the circuit modules is evaluated under 65 nm CMOS technology. The TIA in [1] is adopted, which reported a 10 ns latency and 0.02 pJ energy consumption for each operation. We use the VTC module designed in [2], which shows a latency of 1.17 ns and estimated energy consumption of 0.96 pJ/op according to the formulas of the figure of merit (FOM) and the effective number of bits (ENOB) [3]. Due to the data processing being performed in a pipeline manner, the latency depends on the module that consumes the most. In this case, the latency and energy consumption of analogue readout scheme for one operation are 10 ns and 0.98 pJ, respectively. For the digital readout scheme, both latency and energy consumption during the readout process are dominated by the ADC. An ADC in [4] is used for evaluation, which exhibits a 2.2 ns on latency and 14.3 pJ/op on energy consumption. Based on the above discussion, the analogue readout scheme is more time-consuming, while the digital readout scheme consumes more energy.

**Supplementary Note IV. Equivalent transformation of transpose convolution**

Transpose convolution computing can be expressed as:

$Y=X\text{'}\times W$ (1)

where **Y** and **W** denote the output and kernel matrix, respectively. **X’** is achieved by inserting zero elements between every two elements of the input matrix **X**, which can be written as:

$\text{X}=\left[\begin{matrix} x_{1,1} & x_{1,2} & \ldots\\ x_{2,1} & x_{2,2} & \cdots\\ \vdots& \vdots& \ddots\end{matrix}\right]\to\left[\begin{matrix} 0 & 0 & 0 & 0 & \cdots\\ 0 & x_{1,1} & 0 & x_{1,2} & \cdots\\ 0 & 0 & 0 & 0 & \cdots\\ 0 & x_{2,1} & 0 & x_{2,2} & \cdots\\ \vdots& \vdots& \vdots& \vdots& \ddots\end{matrix}\right]=\text{X'}$ (2)

We unroll the kernel matrix (2×2) as the vector (4×1) so the convolution computing is transformed as vector-matrix multiplication,

$\left[\begin{matrix} y_{2i-1,2j-1} \\ y_{2i-1,2j} \\ y_{2i,2j-1} \\ y_{2i,2j} \end{matrix}\right]=\left[\begin{matrix} 0 & 0 & 0 & x_{i,j} \\ 0 & 0 & x_{i,j} & 0 \\ 0 & x_{i,j} & 0 & 0 \\ x_{i,j} & 0 & 0 & 0 \end{matrix}\right]\left[\begin{matrix} w_{1,1} \\ w_{1,2} \\ w_{2,1} \\ w_{2,2} \end{matrix}\right]$ (3)

The equation (4) can be simplified as:

$\left[\begin{matrix} y_{2i-1,2j-1} \\ y_{2i-1,2j} \\ y_{2i,2j-1} \\ y_{2i,2j} \end{matrix}\right]=x_{i,j}\left[\begin{matrix} w_{2,2} \\ w_{2,1} \\ w_{1,2} \\ w_{1,1} \end{matrix}\right]$ (4)

There are *n* input channels for decoding, then:

$\left[\begin{matrix} y_{2i-1,2j-1} \\ y_{2i-1,2j} \\ y_{2i,2j-1} \\ y_{2i,2j} \end{matrix}\right]=\left[\begin{matrix} w_{_{2,2}}^{c_{1}} & w_{_{2,2}}^{c_{2}} & \cdots& w_{_{2,2}}^{c_{n}} \\ w_{_{2,1}}^{c_{1}} & w_{_{2,1}}^{c_{2}} & \cdots& w_{_{2,1}}^{c_{n}} \\ w_{_{1,2}}^{c_{1}} & w_{_{1,2}}^{c_{2}} & \cdots& w_{_{1,2}}^{c_{n}} \\ w_{_{1,1}}^{c_{1}} & w_{_{1,1}}^{c_{2}} & \cdots& w_{_{1,1}}^{c_{n}} \end{matrix}\right]\left[\begin{matrix} x_{_{i,j}}^{c_{1}} \\ x_{_{i,j}}^{c_{2}} \\ \vdots\\ x_{_{i,j}}^{c_{n}} \end{matrix}\right]$ (5)

The equivalent transformation of transpose convolution is described as equation (5), which can be accelerated by the memristor-based in-memory computing core.

**Supplementary Note V.** **Discussion on memristor multi-bit capabilities boosting system performance**

To discuss how the multi-bit capability of memristor to boost overall efficiency of the proposed storage system (Supplementary Fig. 17c), we evaluated the overheads including latency, energy, and area based on different memristor multi-bit capabilities, and compared with the server-grade central processing unit (CPU)-based processing system (Supplementary Fig. 17a). The VisualQA v2.0 dataset with size 25 GB is used for all the overhead evaluations. Memristors with 2-bit, 4-bit, and 8-bit storage capacity are used to investigate the impact of multi-bit capability on system performance.

**Latency and energy consumption:** We assessed latency and energy consumption during data compression/retrieval by using the storage system demonstrated in the manuscript with different memristor multi-bit capabilities, and compared with those of JPEG, autoencoder (AE), CAE compression techniques implemented in server-grade CPU-based processing system. The circuit metrics used for evaluation could refer to Supplementary Table 2, Table 3, Table 6, and Table 7, and the evaluation method is demonstrated in Methods-System overhead evaluation. Based on these considerations, the comparison results are shown in Supplementary Fig. 18. The results demonstrate that when employing the memristor-based solution for data compression/retrieval, with an increase in memristor’s multi-bit capability (2-bit to 8-bit), the latency and energy consumption overhead decreases by factors from 10× to 24× and 77× to 283×, respectively, compared to server-grade CPU-based solutions.

**Area:** For area evaluation, we consider the area overhead for data storage and encoding/decoding processes. The storage density is discussed based on the area overhead ratio of storing the same amounts of data using server-grade CPU-based processing system and memristor-based near-storage in-memory processing system. All the circuit components in systems are evaluated under 65 nm technology node. For near-storage in-memory processing system, the areas of memristor-based computing bank and storage bank are considered, with the architecture shown in Supplementary Fig. 1. The area metrics of each component could refer to Supplementary Table 1, Table 6, and Table 8, and the evaluation method is elaborated in Methods-Storage density evaluation. In conventional CPU-based processing system, the areas of CPU, dynamic random-access memory (DRAM), and storage are mainly considered, which can also refer to Methods-Storage density evaluation. As a result, the impact of multi-bit capabilities of memristor on areas is shown in Supplementary Fig. 19. It can be calculated that the storage density is improved from 2× (two-layer CAE network with 2-bit memristor) to 18× (six-layer CAE network with 8-bit memristor) according to the areas used before and after compression.

In summary, based on the proposed near-storage in-memory processing system, more storage capacities of memristor could promote the latency and energy efficiencies, and also effectively improve the storage density of the system, showcasing the potential of multi-bit memristors for efficient processing and storing of various data in big data era.

**Supplementary References**

1. Xiao, R. et al. An Energy Efficient Time-Multiplexing Computing-in-Memory Architecture for Edge Intelligence. *IEEE J. Explor. Solid-State Computat.* **8**, 111-118 (2022).
2. Liu, H. et al. A high linear voltage-to-time converter (VTC) with 1.2 V input range for time-domain analog-to-digital converters. *Microelectron. J.* **88**, 18-24 (2019).
3. Osheroff, P. et al. A highly linear 4GS/s uncalibrated voltage-to-time converter with wide input range. In *2016 IEEE International Symposium on Circuits and Systems (ISCAS)* (IEEE, 2016).
4. Tripathi, V. et al. An 8-bit 450-MS/s single-bit/cycle SAR ADC in 65-nm CMOS. in *2013 Proceedings of the ESSCIRC (ESSCIRC)* (IEEE, 2013).
