# Supplementary figures and images for: Memristor-based storage system with convolutional autoencoder-based image compression network
Source: Nat Commun. 2024 Feb 7;15:1132. doi: 10.1038/s41467-024-45312-0 (PMC10850548; doi:10.1038/s41467-024-45312-0)

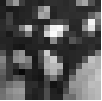

Supplement: Supplementary file 3 — Source data [file 41467_2024_45312_MOESM3_ESM.zip › Source Data File/Figure 4/Images/experimental/compressed feature maps/feature map #1.tif]

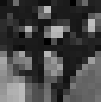

Supplement: Supplementary file 3 — Source data [file 41467_2024_45312_MOESM3_ESM.zip › Source Data File/Figure 4/Images/experimental/compressed feature maps/feature map #2.tif]

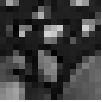

Supplement: Supplementary file 3 — Source data [file 41467_2024_45312_MOESM3_ESM.zip › Source Data File/Figure 4/Images/experimental/compressed feature maps/feature map #3.tif]

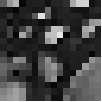

Supplement: Supplementary file 3 — Source data [file 41467_2024_45312_MOESM3_ESM.zip › Source Data File/Figure 4/Images/experimental/compressed feature maps/feature map #4.tif]

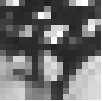

Supplement: Supplementary file 3 — Source data [file 41467_2024_45312_MOESM3_ESM.zip › Source Data File/Figure 4/Images/experimental/compressed feature maps/feature map #5.tif]

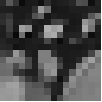

Supplement: Supplementary file 3 — Source data [file 41467_2024_45312_MOESM3_ESM.zip › Source Data File/Figure 4/Images/experimental/compressed feature maps/feature map #6.tif]

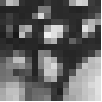

Supplement: Supplementary file 3 — Source data [file 41467_2024_45312_MOESM3_ESM.zip › Source Data File/Figure 4/Images/experimental/compressed feature maps/feature map #7.tif]

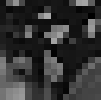

Supplement: Supplementary file 3 — Source data [file 41467_2024_45312_MOESM3_ESM.zip › Source Data File/Figure 4/Images/experimental/compressed feature maps/feature map #8.tif]

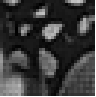

Supplement: Supplementary file 3 — Source data [file 41467_2024_45312_MOESM3_ESM.zip › Source Data File/Figure 4/Images/experimental/ouput maps/output map B.tif]

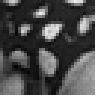

Supplement: Supplementary file 3 — Source data [file 41467_2024_45312_MOESM3_ESM.zip › Source Data File/Figure 4/Images/experimental/ouput maps/output map G.tif]

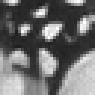

Supplement: Supplementary file 3 — Source data [file 41467_2024_45312_MOESM3_ESM.zip › Source Data File/Figure 4/Images/experimental/ouput maps/output map R.tif]

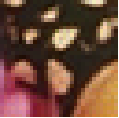

Supplement: Supplementary file 3 — Source data [file 41467_2024_45312_MOESM3_ESM.zip › Source Data File/Figure 4/Images/experimental/ouput maps/output.tif]

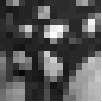

Supplement: Supplementary file 3 — Source data [file 41467_2024_45312_MOESM3_ESM.zip › Source Data File/Figure 4/Images/experimental/pixel quantization/feature map #1.tif]

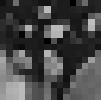

Supplement: Supplementary file 3 — Source data [file 41467_2024_45312_MOESM3_ESM.zip › Source Data File/Figure 4/Images/experimental/pixel quantization/feature map #2.tif]

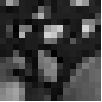

Supplement: Supplementary file 3 — Source data [file 41467_2024_45312_MOESM3_ESM.zip › Source Data File/Figure 4/Images/experimental/pixel quantization/feature map #3.tif]

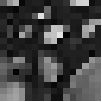

Supplement: Supplementary file 3 — Source data [file 41467_2024_45312_MOESM3_ESM.zip › Source Data File/Figure 4/Images/experimental/pixel quantization/feature map #4.tif]

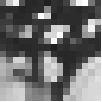

Supplement: Supplementary file 3 — Source data [file 41467_2024_45312_MOESM3_ESM.zip › Source Data File/Figure 4/Images/experimental/pixel quantization/feature map #5.tif]

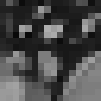

Supplement: Supplementary file 3 — Source data [file 41467_2024_45312_MOESM3_ESM.zip › Source Data File/Figure 4/Images/experimental/pixel quantization/feature map #6.tif]

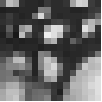

Supplement: Supplementary file 3 — Source data [file 41467_2024_45312_MOESM3_ESM.zip › Source Data File/Figure 4/Images/experimental/pixel quantization/feature map #7.tif]

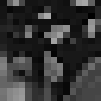

Supplement: Supplementary file 3 — Source data [file 41467_2024_45312_MOESM3_ESM.zip › Source Data File/Figure 4/Images/experimental/pixel quantization/feature map #8.tif]

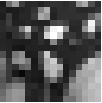

Supplement: Supplementary file 3 — Source data [file 41467_2024_45312_MOESM3_ESM.zip › Source Data File/Figure 4/Images/simulation/compressed feature maps/feature map #1.tif]

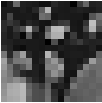

Supplement: Supplementary file 3 — Source data [file 41467_2024_45312_MOESM3_ESM.zip › Source Data File/Figure 4/Images/simulation/compressed feature maps/feature map #2.tif]

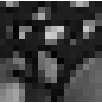

Supplement: Supplementary file 3 — Source data [file 41467_2024_45312_MOESM3_ESM.zip › Source Data File/Figure 4/Images/simulation/compressed feature maps/feature map #3.tif]

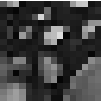

Supplement: Supplementary file 3 — Source data [file 41467_2024_45312_MOESM3_ESM.zip › Source Data File/Figure 4/Images/simulation/compressed feature maps/feature map #4.tif]

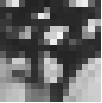

Supplement: Supplementary file 3 — Source data [file 41467_2024_45312_MOESM3_ESM.zip › Source Data File/Figure 4/Images/simulation/compressed feature maps/feature map #5.tif]

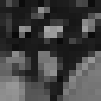

Supplement: Supplementary file 3 — Source data [file 41467_2024_45312_MOESM3_ESM.zip › Source Data File/Figure 4/Images/simulation/compressed feature maps/feature map #6.tif]

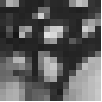

Supplement: Supplementary file 3 — Source data [file 41467_2024_45312_MOESM3_ESM.zip › Source Data File/Figure 4/Images/simulation/compressed feature maps/feature map #7.tif]

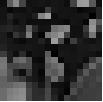

Supplement: Supplementary file 3 — Source data [file 41467_2024_45312_MOESM3_ESM.zip › Source Data File/Figure 4/Images/simulation/compressed feature maps/feature map #8.tif]

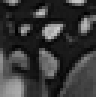

Supplement: Supplementary file 3 — Source data [file 41467_2024_45312_MOESM3_ESM.zip › Source Data File/Figure 4/Images/simulation/output maps/output map B.tif]

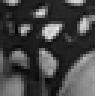

Supplement: Supplementary file 3 — Source data [file 41467_2024_45312_MOESM3_ESM.zip › Source Data File/Figure 4/Images/simulation/output maps/output map G.tif]

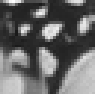

Supplement: Supplementary file 3 — Source data [file 41467_2024_45312_MOESM3_ESM.zip › Source Data File/Figure 4/Images/simulation/output maps/output map R.tif]

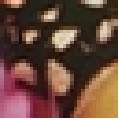

Supplement: Supplementary file 3 — Source data [file 41467_2024_45312_MOESM3_ESM.zip › Source Data File/Figure 4/Images/simulation/output maps/output.tif]
